# Association of Haemostatic and Inflammatory Biomarkers with Nephropathy in Type 1 Diabetes Mellitus

**DOI:** 10.1155/2016/2315260

**Published:** 2015-12-06

**Authors:** Caroline Pereira Domingueti, Rodrigo Bastos Fóscolo, Janice Sepúlveda Reis, Fernanda Magalhães Freire Campos, Luci Maria S. Dusse, Maria das Graças Carvalho, Karina Braga Gomes, Ana Paula Fernandes

**Affiliations:** ^1^Departamento de Analises Clínicas e Toxicológicas, Faculdade de Farmácia, Universidade Federal de São João Del-Rei, Campus Centro Oeste Dona Lindu, 35501-296 Divinopolis, MG, Brazil; ^2^Departamento de Clínica Médica, Faculdade de Medicina, Universidade Federal de Minas Gerais, 30130-100 Belo Horizonte, MG, Brazil; ^3^Departamento de Endocrinologia e Metabolismo, Instituto de Educação e Pesquisa da Santa Casa de Belo Horizonte, 30150-250 Belo Horizonte, MG, Brazil; ^4^Departamento de Analises Clínicas e Toxicológicas, Faculdade de Farmácia, Universidade Federal de Minas Gerais, 31270-901 Belo Horizonte, MG, Brazil

## Abstract

This study aimed at investigating the association between haemostatic biomarkers, proinflammatory, and anti-inflammatory cytokines with chronic kidney disease in type 1 diabetic patients. Patients were divided into two groups: with nephropathy (albuminuria ≥ 30 mg/g and/or GFR < 60 mL/min/1.73 m^2^), *n* = 65; and without nephropathy (albuminuria < 30 mg/g and GFR ≥ 60 mL/min/1.73 m^2^), *n* = 60. INF-*γ*, IL-6, IL-10, and TNF-*α* plasma levels were determined by flow cytometry. VWF, ADAMTS13 antigen, and D-Dimer plasma levels were determined by enzyme-linked immunosorbent assay and ADAMTS13 activity was assessed by fluorescence resonance energy transfer assay. Elevated levels of INF-*γ*, VWF, ADAMTS13 antigen, D-Dimer, and reduced ADAMTS13 activity/antigen ratio were observed in patients with nephropathy as compared to those without nephropathy (*P* = 0.001, *P* < 0.001, *P* < 0.001, *P* < 0.001, and *P* < 0.001, resp.). Cytokines and haemostatic biomarkers remained associated with nephropathy after adjustments (use of statin, acetylsalicylic acid, angiotensin converting enzyme inhibitor, and angiotensin antagonist). INF-*γ*, TNF-*α*, and IL-10 significantly correlated with haemostatic biomarkers. Inflammatory and hypercoagulability status are associated with nephropathy in type 1 diabetes mellitus and an interrelationship between them may play an important role in pathogenesis of diabetic nephropathy.

## 1. Introduction

Chronic kidney disease (CKD) is a very common complication of diabetes mellitus (DM) [1]. It is defined as the presence of abnormalities in renal structure or function for longer than three months, with implications to healthy. The diagnostic criteria for CKD consist of the presence of one or more biomarkers of kidney parenchyma injury such as albuminuria and/or a glomerular filtration rate (GFR) lower than 60 mL/min/1.73 m^2^ over longer than three months [2].

The complications of diabetic nephropathy, such as end stage renal disease and cardiovascular events, are responsible for an increased morbidity and mortality among diabetic patients [3]. There are few available pharmacological therapies for treatment of renal disease [4]. Therefore, it is very important to study molecular and metabolic mechanisms involved with the development and progression of renal dysfunction in DM, since this could contribute to the development of new therapeutic strategies.

Emerging evidence suggests that inflammatory pathways play an important role in the development and progression of diabetic nephropathy. It has been reported that T cells and inflammatory cytokines exert central roles in the development of renal disease in DM [5]. Moreover, hyperglycemia can induce the expression of several proinflammatory cytokines, such as interferon gamma (INF-*γ*), interleukin-6 (IL-6), and tumor necrosis factor-alpha (TNF-*α*), leading to the development of a chronic subclinical inflammatory status in DM [6]. The deficiency of interleukin-10 (IL-10), an anti-inflammatory cytokine, may also be associated with this increased inflammatory status [7].

The interrelation between inflammation and metabolic abnormalities in DM may lead to endothelial injury and the release of von Willebrand factor (VWF) [6]. Increased levels of VWF, a biomarker of endothelial dysfunction, have been associated with renal disease in type 1 (DM1) and type 2 diabetes mellitus (DM2) [8, 9]. VWF promotes platelet adhesion at vascular damage sites, where it mediates the progression of thrombus formation [10]. Elevated levels of VWF can promote microthrombi formation in vasculature, leading to hypercoagulability status and formation of D-Dimer, which is a fragment of fibrin degradation and has also been associated with diabetic nephropathy [11, 12]. ADAMTS13 (a disintegrin and metalloproteinase with thrombospondin type 1 motif, member 13) is an enzyme responsible for cleavage of large multimers of VWF [10]. An imbalance between VWF and ADAMTS13 may also contribute to the development of microvascular complications in diabetic patients [13].

In a previous study, we have described the association between VWF, ADAMTS13, and D-Dimer with different levels of renal dysfunction in DM1 patients and we have raised the hypothesis that inflammation could be associated with hypercoagulability in patients with diabetic nephropathy [14]. Therefore, the aim of the present study was to investigate the association between the haemostatic biomarkers VWF, ADAMTS13, and D-Dimer, the proinflammatory cytokines INF-*γ*, IL-6, and TNF-*α*, and the anti-inflammatory cytokine IL-10 with CKD in DM1 patients.

## 2. Patients and Methods

### 2.1. Ethics

All procedures performed in this study were in accordance with the 1964 Declaration of Helsinki and its later amendments or comparable ethical standards. This study was approved by the Research Ethics Committee of Federal University of Minas Gerais (CAAE, 0392.0.203.000-11) and informed consent was obtained from all individual participants included in the study. The research protocol did not interfere with any medical recommendations or prescriptions.

### 2.2. Patients

The clinical records of all 240 DM1 patients receiving assistance at Endocrinology Outpatient Services of the University Hospital (*Hospital das Clínicas*) and Santa Casa/Belo Horizonte, Brazil, from November 2011 to September 2012, were analyzed. After application of exclusion criteria, 125 patients with clinical and laboratorial diagnosis of DM1, according to American Diabetes Association criteria [15], from 18 to 60 years of age, were selected for this study.

DM1 patients with hepatic disease, alcoholism, haemostatic abnormalities, malignant diseases, acute infectious, pregnancy, undergoing hemodialysis, and history of kidney transplantation and cardiovascular diseases were excluded from the study.

### 2.3. Study Protocol

A detailed history and clinical variables of each patient were obtained from medical records: age, sex, body mass index (BMI), diabetes duration, presence of diabetes complications such as retinopathy and neuropathy, and use of medicines such as angiotensin converting enzyme inhibitor (ACEi), angiotensin antagonist, thyroxine, statin, and acetylsalicylic acid (AAS).

DM1 patients were divided into two groups: with CKD (albuminuria ≥ 30 mg/g and/or GFR < 60 mL/min/1.73 m^2^), *n* = 65; and without CKD (albuminuria < 30 mg/g and GFR ≥ 60 mL/min/1.73 m^2^), *n* = 60. The presence of increased albuminuria and reduced GFR was confirmed in two out of three occasions, over a period between three and six months [2].

### 2.4. Determination of Biochemistry Parameters

HbA1c was determined by immunoturbidimetric method in EDTA whole blood samples and creatinine was determined by enzymatic method in serum samples, using Johnson & Johnson dry chemistry technology kits (Ortho Clinical Diagnostics) and VITROS 4600 analyser. The GFR was estimated using the Chronic Kidney Disease Epidemiology Collaboration (CKD-EPI) formula based on creatinine [16].

Urinary albumin excretion (UAE) was determined in urine samples collected after at least 4 hours of urinary retention in the morning, and urinary albumin was normalized by urinary creatinine. Urinary albumin was evaluated by immunoturbidimetric method and urinary creatinine was assessed by enzymatic method, using Johnson & Johnson dry chemistry technology kits (Ortho Clinical Diagnostics) and VITROS 4600 analyser.

### 2.5. Inflammatory Cytokines Measurement

INF-*γ*, IL-6, IL-10, and TNF-*α* plasma levels were determined by flow cytometry, using* Human Basic Kit FlowCytomix* (eBioscience), following the manufacturer's recommendations. Data acquisition and analysis were performed in dual-laser FACS caliber TM flow cytometer (BD Biosciences Pharmingen, San Jose, CA, USA), using the BD Bioscience CBA software.

### 2.6. Haemostatic Biomarkers Measurement

VWF, ADAMTS13 antigen, and D-Dimer plasma levels were determined by enzyme-linked immunosorbent assay (ELISA), using IMUBIND VWF kit (American Diagnostica), IMUBIND ADAMTS13 kit (American Diagnostica), and ASSERACHROM D-Di kit (StagoDiagnostica), respectively. ADAMTS13 activity was assessed by fluorescence resonance energy transfer (FRET) assay, using ACTIFLUOR ADAMTS13 activity kit (Sekisui Diagnostics). Intra- and interassay coefficients of variations were, respectively, 9% and 13% for VWF, 4.0% and 7.3% for ADAMYS13 antigen, <6% and <10% for D-Dimer, and 4.1% and 4.4% for ADAMTS13 activity.

### 2.7. Statistical Analysis

Statistical comparisons were performed using SPSS software (version 20.0, SPSS). Shapiro-Wilk test was used to test if continuous variables were normally distributed. Data normally distributed were expressed as mean ± SD and were compared by Student's *t*-test. Data not normally distributed were expressed as median (percentiles 25%–75%) and were compared by Mann-Whitney *U* test. Categorical variables were expressed as frequencies and compared using chi-square test (*χ* ^2^). Association between haemostatic and inflammatory biomarkers with CKD was evaluated by bivariate and multivariate logistic regression analysis and odds ratio was calculated. Variables included in multivariate logistic regression analysis were previously associated with CKD in bivariate logistic regression analysis (*P* < 0.2) and consisted of use of ACEi or angiotensin antagonist, use of statin, and use of AAS. Correlations between proinflammatory cytokines and the anti-inflammatory ones and between inflammatory and haemostatic biomarkers were performed using Spearman correlation test. Differences were considered significant when *P* ≤ 0.05.

## 3. Results

Characteristics and clinical variables of 125 DM1 patients included in this cross-sectional study are presented in Table 1. Patients with CKD had lower BMI (*P* = 0.030) and a higher frequency of use of IECA or angiotensin antagonist (*P* < 0.001), statin (*P* < 0.001), and AAS (*P* = 0.001) than patients without CKD. There were no significant differences between the groups regarding age, sex, diabetes duration, HbA1c, use of thyroxine, and presence of neuropathy. However, a higher frequency of retinopathy in group of patients with nephropathy as compared to patients without CKD (*P* < 0.001) was observed.

INF-*γ*, TNF-*α*, IL6, and IL-10 plasma levels were higher in patients with CKD as compared to those without nephropathy (*P* = 0.001, *P* = 0.004, *P* = 0.016, and *P* = 0.030, resp.) (Table 1). Patients with CKD also presented elevated plasma levels of VWF, ADAMTS13 antigen, ADAMTS13 activity, D-Dimer and VWF/ADAMTS13 activity ratio, and a reduced ADAMTS13 activity/antigen ratio than those without nephropathy (*P* < 0.001, *P* < 0.001, *P* = 0.003, *P* < 0.001, *P* = 0.006, and *P* < 0.001, resp.). VWF/ADAMTS13 antigen ratio was not significantly different between the groups.

The results of the bivariate logistic regression analysis are presented in Table 2. In general, inflammatory and haemostatic biomarkers were significant associated with CKD, corresponding to OR of 1.021 (1.008–1.035) for INF-*γ*, 1.006 (1.002–1.011) for TNF-*α*, 1.224 (1.051–1.426) for IL-6, 1.002 (1.000–1.003) for IL-10, 1.003 (1.001–1.004) for VWF, 1.005 (1.002–1.007) for ADAMTS13 antigen, 1.030 (1.009–1.051) for ADAMTS13 activity, 0.667 (0.490–0.907) for VWF/ADAMTS13 antigen, 1.129 (1.031–1.236) for VWF/ADAMTS13 activity, 7 × 10^−11^ (4 × 10^−15^–1 × 10^−5^) for ADAMTS13 activity/antigen, and 1.008 (1.004–1.012) for D-Dimer. These associations remained significant even after adjustment for use of ACEi or angiotensin antagonist, statin, and AAS, as shown by multivariate regression logistic analysis (Table 2).

**Figure 1:**
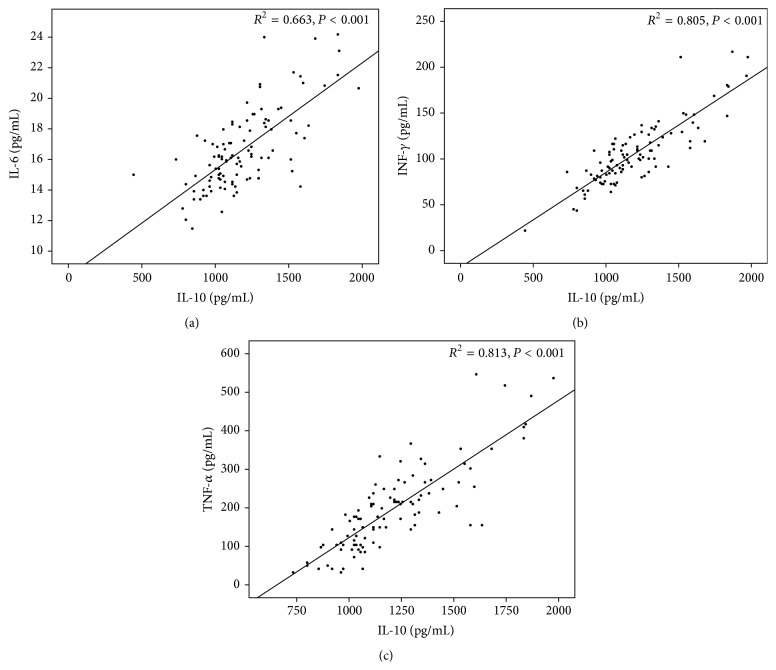
Correlation between IL-6 (a), INF-*γ* (b), and TNF-*α* (c) with IL-10 in type 1 diabetic patients.

All proinflammatory cytokines IL-6, INF-*γ*, and TNF-*α* presented a significant correlation (*P* < 0.001) with the anti-inflammatory cytokine IL-10 (*R* ^2^ = 0.689, 0.805, and 0.813, resp.), as presented in Figure 1. Correlations between inflammatory and haemostatic biomarkers are presented in Table 3. INF-*γ* presented a significant correlation with VWF, ADAMTS13 antigen, VWF/ADAMTS13 activity ratio, and ADAMTS13 activity/antigen ratio (*R* = 0.264, 0.192, 0.220, and −0.385, resp.). TNF-*α* was significantly correlated with ADAMTS13 antigen, ADAMTS13 activity/antigen ratio, and D-Dimer (*R* = 0.291, −0.337, and 0.217, resp.). IL-10 showed a significant correlation with VWF, VWF/ADAMTS13 activity ratio, ADAMTS13 activity/antigen ratio, and D-Dimer (*R* = 0.212, 0.192, −0.270, and 0.244, resp.). IL-6 was not significantly correlated with none of the haemostatic biomarkers.

## 4. Discussion

In our study, higher levels of INF-*γ*, IL-6, and TNF-*α* were observed in DM1 patients with CKD in comparison to those without nephropathy, which is in agreement with other studies [17–22].

Hyperglycemia is associated with increased production of advanced glycation end-products (AGEs), which can bind to their receptors present on the surface of endothelial cells, smooth muscle cells, fibroblasts, lymphocytes, monocytes, and macrophages, resulting in activation of NF-*κ*B which can enhance INF-*γ* transcription in T cells and IL-6 and TNF-*α* transcription in diabetic glomerulus [23–25]. Large amounts of INF-*γ* are produced by T helper 1 (Th1) cells, promoting activation of macrophages and cell-mediated immunity, which may mediate tissue injury in diabetic patients [26]. TNF-*α* can induce monocyte chemoattractant protein-1 (MCP-1) expression in mesangial cells, resulting in macrophage recruitment and accumulation in glomerulus, exacerbating kidney inflammation [27]. Hyperglycemia also causes enhanced cycle oxygenize-2 (COX-2) expression and prostaglandin E2 (PGE2) production, which induces IL-6 expression in tubular epithelial cells, contributing to the development of glomerulosclerosis, interstitial fibrosis, and albuminuria [28]. Furthermore, IL-6 and TNF-*α* are cytotoxic cytokines and may contribute to glomerulus endothelial cells damage, which can stimulate the release and expression of procoagulant molecules, such as VWF, plasminogen activator inhibitor-1 (PAI-1), and tissue factor. They can also inhibit the expression of anticoagulant molecules, such as thrombomodulin, by endothelial cells [29], resulting in hypercoagulability state, which may also contribute to progression of diabetic nephropathy [8].

Wolkow et al. [30] reported that IL-6 may be associated with the progression of nephropathy in DM1 patients with microalbuminuria and a meta-analysis has demonstrated that IL-6 is a valid biomarker predicting the progression of nephropathy in DM2 [31]. Moreover, urinary TNF-*α* and soluble TNF receptors 1 and 2 (TNFR1 and TNFR2) have been shown to be significant predictors of GFR decline in DM2 patients [32, 33]. Therefore, it is possible to suggest that hyperglycemia promotes the development of a chronic low grade inflammation in renal parenchyma, characterized by increased levels of proinflammatory cytokines INF-*γ*, TNF-*α*, and IL-6 levels, which may be involved in the pathogenesis of diabetic nephropathy.

IL-10 is produced by T helper 2 (Th2) cells and plays an important role in limiting the cascade of proinflammatory cytokines activation and downregulating T cell-mediated immune responses [34]. Treatment of rats with IL-10 reduced proteinuria and inflammatory status and attenuated renal injury [35]. A lower frequency of CG genotype polymorphism IL-10 -1082G/A, which is related to higher expression of this cytokine, was associated with nephropathy in DM2 [36]. In our study, patients with CKD presented higher levels of IL-10 than patients without nephropathy, which is in agreement with other study [34]. This result could be partially explained by a compensatory mechanism, in which IL-10 plasma levels increase as a consequence of higher plasma levels of proinflammatory cytokines INF-*γ*, TNF-*α*, and IL-6 in patients with nephropathy to regulate the inflammatory status, since IL-10 is an anti-inflammatory cytokine. The significant correlation between proinflammatory cytokines and IL-10 observed in this study may corroborate this hypothesis. This compensatory increase in IL-10 levels could also prolong the course of nephropathy in DM1 patients.

Elevated plasma levels of VWF were associated with diabetic nephropathy in the present study, which was also verified by other authors [9, 37]. It was demonstrated that some inflammatory cytokines, such as INF-*γ*, IL-6, and TNF-*α*, can promote the release of VWF [29, 38]. Indeed, a significant correlation between VWF and INF-*γ* was observed in this study, which indicates that the interrelation between inflammation and endothelial dysfunction may be involved in the pathogenesis of diabetic nephropathy.

ADAMTS13 antigen and activity plasma levels were also elevated in DM1 patients with CKD as compared to those without renal dysfunction, which was also found in other studies [39]. This result may be explained by a compensatory mechanism, by which ADAMTS13 synthesis is increased due to the marked elevation in VWF plasma levels, keeping VWF/ADAMTS13 antigen ratio unchanged. However, the rise in ADAMTS13 antigen levels was not accompanied by a proportional increase in ADAMTS13 activity, since ADAMTS13 activity/ADAMTS13 Ag ratio was lower and VWF/ADAMTS13 activity ratio was higher in DM1 patients with nephropathy.

An* in vitro* study has demonstrated that IL-6 can inhibit ADAMTS13 activity [40], compromising the proteolysis of VWF. TNF-*α* and INF-*γ* presented a significant negative correlation with ADAMTS13 activity/antigen ratio and a significant positive correlation with ADAMTS13 antigen, but not with ADAMTS13 activity, which is in agreement with the hypothesis that the inflammatory status induced by hyperglycemia may also result in reduced activity of ADAMTS13 and, consequently, in reduced proteolysis of VWF, leading to an imbalance between VWF and ADAMTS13 activity in DM1 patients with CKD. This imbalance may promote microthrombi formation and hypercoagulability status, resulting in elevated D-Dimer plasma levels, which was also observed in DM1 with CKD in this study and other studies [11, 12]. A significant positive correlation between D-Dimer and TNF-*α* observed in the present study reinforces the link between inflammation and hypercoagulability.

Increased proinflammatory cytokines plasma levels have been associated with the development of cardiovascular disease in DM1 patients [21]. As these cytokines can promote the release of procoagulant molecules, such as VWF [29], and possibly can reduce ADAMTS13 activity, promoting an imbalance between VWF and ADAMTS13 activity and, consequently, microthrombi formation, it is possible to suggest that the chronic low grade inflammation present in patients with CKD may be, at least partially, responsible for the link between diabetic nephropathy and high risk of cardiovascular outcomes. However, longitudinal studies are still necessary to elucidate this issue.

In conclusion, increased plasma levels of proinflammatory cytokines INF-*γ*, TNF-*α*, and IL-6, anti-inflammatory cytokine IL-10, and haemostatic biomarkers VWF, ADAMTS13 antigen, ADAMTS13 activity, and D-Dimer are associated with CKD in DM1 patients, and the imbalance between VWF/ADAMTS13 activity and ADAMTS13 activity/antigen is correlated with inflammatory cytokines, suggesting that an interrelation between inflammation and hypercoagulability may contribute to the development and progression of renal disease in DM1. These findings may be useful for guiding future longitudinal studies aiming at a better comprehension of the interrelation between inflammation and hypercoagulability with diabetic nephropathy progression, enabling the identification of new biomarkers of renal disease progression and the introduction of new therapeutic strategies.

## Figures and Tables

**Table 1:** Characteristics of type 1 diabetic patients with and without chronic kidney disease.

	Patients without CKD	Patients with CKD	*P*
Number of individuals (*n*)	60	65	
Age (years)	32 (25–37)	34 (27–43)	NS
Sex/male (*n*, %)	23 (38.3)	22 (33.8)	NS
BMI (kg/m^2^)	24 ± 3	23 ± 2	*P* = 0.030
Diabetes duration (years)	18 ± 8	19 ± 6	NS
Retinopathy (*n*, %)	16 (26.7)	39 (60.0)	*P* < 0.001
Neuropathy (*n*, %)	11 (18.3)	9 (13.8)	NS
Use of ACEi or angiotensin antagonist (*n*, %)	29 (48.3)	44 (67.7)	*P* < 0.001
Use of statin (*n*, %)	10 (16.7)	30 (46.2)	*P* < 0.001
Use of AAS (*n*, %)	3 (5.0)	18 (27.7)	*P* = 0.001
Use of thyroxine (*n*, %)	6 (10.0)	12 (18.5)	NS
HbA1c (%)	8.4 ± 1.6	8.7 ± 1.4	NS
Creatinine (mg/dL)	0.79 (0.66–0.88)	1.07 (0.76–1.49)	*P* < 0.001
UAE (mg/g of creatinine)	6 (4–14)	65 (38–141)	*P* < 0.001
GFR (mL/min/1.73 m^2^)	114 (104–123)	75 (44–106)	*P* < 0.001
INF-*γ* (pg/mL)	95 ± 20	119 ± 45	*P* = 0.001
TNF-*α* (pg/mL)	166 (104–215)	215 (149–314)	*P* = 0.004
IL-6 (pg/mL)	16 (14–18)	17 (15–19)	*P* = 0.016
IL-10 (pg/mL)	1106 (1019–1295)	1236 (1024–1523)	*P* = 0.030
VWF (mU/mL)	1028 ± 287	1350 ± 414	*P* < 0.001
ADAMTS13 antigen (ng/mL)	305 (231–509)	549 (357–638)	*P* < 0.001
ADAMTS13 activity (%)	95 ± 16	105 ± 21	*P* = 0.003
VWF/ADAMTS13 antigen	3.1 (1.9–4.2)	2.6 (1.9–3.3)	NS
VWF/ADAMTS13 activity	11.1 ± 3.8	13.2 ± 4.4	*P* = 0.006
ADAMTS13 activity/antigen	0.31 (0.20–0.40)	0.18 (0.17–0.19)	*P* < 0.001
D-Dimer (ng/mL)	191 (137–258)	309 (202–451)	*P* < 0.001

Normally distributed data were expressed as mean ± SD and compared by *t*-test. Not normally distributed data were expressed as median (percentiles 25%–75%) and compared by Mann-Whitney *U* test. Categorical variables were expressed as frequencies *n* (%) and compared using the chi-square test (*χ* ^2^). Body mass index (BMI), time of diagnosis, HbA1c, interferon gamma (INF-*γ*), von Willebrand factor (VWF), ADAMTS13 activity, and VWF/ADAMTS13 activity ratio were normally distributed. Age, creatinine, urinary albumin excretion (UAE), glomerular filtration rate (GFR), tumor necrosis factor-alpha (TNF-*α*), interleukin-6 (IL-6), interleukin-10 (IL-10), ADAMTS13 antigen, VWF/ADAMTS13 antigen ratio, ADAMTS13 activity/antigen ratio, and D-Dimer were not normally distributed. NS = not significant. CKD = chronic kidney disease. AAS = acetylsalicylic acid. ACEi = angiotensin converting enzyme inhibitor.

**Table 2:** Association between haemostatic and inflammatory biomarkers with chronic kidney disease in type 1 diabetic patients.

Variable	Odds ratio (95% confidence interval) unadjusted	*P*	Odds ratio (95% confidence interval) adjusted	*P*
INF-*γ*	1.021 (1.008–1.035)	0.002	1.025 (1.010–1.040)	0.001
TNF-*α*	1.006 (1.002–1.011)	0.002	1.008 (1.003–1.013)	0.001
IL-6	1.224 (1.051–1.426)	0.009	1.304 (1.091–1.559)	0.004
IL-10	1.002 (1.000–1.003)	0.018	1.003 (1.001–1.004)	0.003
VWF	1.003 (1.001–1.004)	<0.001	1.003 (1.001–1.004)	<0.001
ADAMTS13 antigen	1.005 (1.002–1.007)	<0.001	1.005 (1.002–1.007)	<0.001
ADAMTS13 activity	1.030 (1.009–1.051)	0.004	1.034 (1.011–1.057)	0.004
VWF/ADAMTS13 antigen	0.667 (0.490–0.907)	0.010	0.702 (0.501–0.984)	0.040
VWF/ADAMTS13 activity	1.129 (1.031–1.236)	0.009	1.125 (1.017–1.244)	0.022
ADAMTS13 activity/antigen	7 × 10^−11^ (4 × 10^−16^–1 × 10^−5^)	<0.001	7 × 10^−11^ (4 × 10^−16^–1 × 10^−5^)	<0.001
D-Dimer	1.008 (1.004–1.012)	<0.001	1.008 (1.004–1.012)	<0.001

Data was evaluated by bivariate and multivariate logistic regression analysis and are presented as odds ratio (95% confidence interval) per unit increase of exposure variable. Variables included in multivariate logistic regression analysis were previously associated with chronic kidney disease in bivariate logistic regression analysis (*P* < 0.2) and consisted of use of angiotensin converting enzyme inhibitor (ACEi) or angiotensin antagonist, use of statin, and use of acetylsalicylic acid (AAS).

**Table 3:** Correlations between haemostatic and inflammatory biomarkers in type 1 diabetic patients.

	VWF	ADAMTS13 antigen	ADAMTS13 activity	VWF/ADAMTS13 antigen	VWF/ADAMTS13 activity	ADAMTS13 activity/antigen	D-Dimer
INF-*γ*	0.264^*∗∗*^	0.192^*∗*^	0.046	−0.125	0.220^*∗∗*^	−0.385^*∗∗*^	0.105
TNF-*α*	0.183	0.291^*∗∗*^	0.127	−0.158	0.073	−0.337^*∗∗*^	0.217^*∗*^
IL-6	0.082	0.083	−0.007	−0.044	0.069	−0.130	0.162
IL-10	0.212^*∗*^	0.103	0.013	−0.003	0.192^*∗*^	−0.270^*∗*^	0.244^*∗*^

Correlations were performed using Spearman correlation test. Data was expressed as correlation coefficient (*R*).

^*∗*^Correlation is significant at the 0.05 level. ^*∗∗*^Correlation is significant at the 0.01 level.

## References

[1] Yamagishi S.-I., Matsui T. (2010). Advanced glycation end products, oxidative stress and diabetic nephropathy. *Oxidative Medicine and Cellular Longevity*.

[2] National Disease: Improving Global Outcomes (KDIGO) CKD Work Group (2013). KDIGO clinical practive guideline for the evaluation and management of chronic kidney disease. *Kidney International Supplements*.

[3] Kanasaki K., Taduri G., Koya D. (2013). Diabetic nephropathy: the role of inflammation in fibroblast activation and kidney fibrosis. *Frontiers in Endocrinology*.

[4] Duran-Salgado M. B., Rubio-Guerra A. F. (2014). Diabetic nephropathy and inflammation. *World Journal of Diabetes*.

[5] Navarro-González J. F., Mora-Fernández C., De Fuentes M. M., García-Pérez J. (2011). Inflammatory molecules and pathways in the pathogenesis of diabetic nephropathy. *Nature Reviews Nephrology*.

[6] Goldberg R. B. (2009). Cytokine and cytokine-like inflammation markers, endothelial dysfunction, and imbalanced coagulation in development of diabetes and its complications. *The Journal of Clinical Endocrinology & Metabolism*.

[7] Trifunović J., Miller L., Debeljak Ž., Horvat V. (2015). Pathologic patterns of interleukin 10 expression—a review. *Biochemia Medica*.

[8] Kessler L., Wiesel M. L., Attali P., Mossard J. M., Cazenave J. P., Finget M. (1998). Von willebrand factor in diabetic angiopathy. *Diabetes and Metabolism*.

[9] Chan N. N., Fuller J. H., Rubens M., Colhoun H. M. (2003). Von Willebrand factor in type 1 diabetes: its relationship with endothelial nitric oxide production and coronary artery calcification. *Medical Science Monitor*.

[10] Jenkins P. V., O'Donnell J. S. (2006). ABO blood group determines plasma von Willebrand factor levels: a biologic function after all?. *Transfusion*.

[11] El.Asrar M. A., Adly A. A., El Hadidy E. S., Abdelwahab M. A. (2012). D-dimer levels in type 1 and type 2 diabetic children and adolescents; relation to microvascular complications and dyslipidemia “own data and review”. *Pediatric Endocrinology Reviews*.

[12] Long Z. F., Qu G. Y., Xu M. (2001). Relationship between the level of plasma D-dimer and diabetic microangiopathy. *Hunan Yi Ke Da Xue Xue Bao*.

[13] Domingueti C. P., Dusse L. M. S., Carvalho M. D. G., Gomes K. B., Fernandes A. P. (2013). Hypercoagulability and cardiovascular disease in diabetic nephropathy. *Clinica Chimica Acta*.

[14] Domingueti C. P., Dusse L. M., Fóscolo R. B. (2015). Von Willebrand factor, ADAMTS13 and D-Dimer are correlated with different levels of nephropathy in type 1 diabetes mellitus. *PLoS ONE*.

[15] American Diabetes Association (2015). Standards of medical care in diabetes. *Diabetes Care*.

[16] Levey A. S., Stevens L. A., Schmid C. H. (2009). A new equation to estimate glomerular filtration rate. *Annals of Internal Medicine*.

[17] Zhang C., Xiao C., Wang P. (2014). The alteration of Th1/Th2/Th17/Treg paradigm in patients with type 2 diabetes mellitus: relationship with diabetic nephropathy. *Human Immunology*.

[18] Nosratabadi R., Arababadi M. K., Hassanshahi G. (2009). Evaluation of IFN-*γ* serum level in nephropatic type 2 diabetic patients. *Pakistan Journal of Biological Sciences*.

[19] Shelbaya S., Amer H., Seddik S. (2012). Study of the role of interleukin-6 and highly sensitive C-reactive protein in diabetic nephropathy in type 1 diabetic patients. *European Review for Medical and Pharmacological Sciences*.

[20] Sahakyan K., Klein B. E. K., Lee K. E., Tsai M. Y., Klein R. (2010). Inflammatory and endothelial dysfunction markers and proteinuria in persons with type 1 diabetes mellitus. *European Journal of Endocrinology*.

[21] Schram M. T., Chaturvedi N., Schalkwijk C. G., Fuller J. H., Stehouwer C. D. A. (2005). Markers of inflammation are cross-sectionally associated with microvascular complications and cardiovascular disease in type 1 diabetes—the EURODIAB Prospective Complications Study. *Diabetologia*.

[22] Saraheimo M., Teppo A.-M., Forsblom C., Fagerudd J., Groop P.-H. (2003). Diabetic nephropathy is associated with low-grade inflammation in type 1 diabetic patients. *Diabetologia*.

[23] Ramakrishnan P., Clark P. M., Mason D. E., Peters E. C., Hsieh-Wilson L. C., Baltimore D. (2013). Activation of the transcriptional function of the NF-kB protein c-Rel by *O*-GlcNAc glycosylation. *Science Signaling*.

[24] Giannini C., Mohn A., Chiarelli F., Kelnar C. J. H. (2011). Macrovascular angiopathy in children and adolescents with type 1 diabetes. *Diabetes/Metabolism Research and Reviews*.

[25] Hasegawa G., Nakano K., Sawada M. (1991). Possible role of tumor necrosis factor and interleukin-1 in the development of diabetic nephropathy. *Kidney International*.

[26] Wu C.-C., Chen J.-S., Lu K.-C. (2010). Aberrant cytokines/chemokines production correlate with proteinuria in patients with overt diabetic nephropathy. *Clinica Chimica Acta*.

[27] Matoba K., Kawanami D., Ishizawa S., Kanazawa Y., Yokota T., Utsunomiya K. (2010). Rho-kinase mediates TNF-*α*-induced MCP-1 expression via p38 MAPK signaling pathway in mesangial cells. *Biochemical and Biophysical Research Communications*.

[28] Mohamed R., Jayakumar C., Ramesh G. (2013). Chronic administration of EP4-selective agonist exacerbates albuminuria and fibrosis of the kidney in streptozotocin-induced diabetic mice through IL-6. *Laboratory Investigation*.

[29] Margetic S. (2012). Inflammation and haemostasis. *Biochemia Medica*.

[30] Wolkow P. P., Niewczas M. A., Perkins B. (2008). Association of urinary inflammatory markers and renal decline in microalbuminuric type 1 diabetics. *Journal of the American Society of Nephrology*.

[31] Hellemons M. E., Kerschbaum J., Bakker S. J. L. (2012). Validity of biomarkers predicting onset or progression of nephropathy in patients with type 2 diabetes: a systematic review. *Diabetic Medicine*.

[32] Wu J., Ding Y., Zhu C. (2013). Urinary TNF-*α* and NGAL are correlated with the progression of nephropathy in patients with type 2 diabetes. *Experimental and Therapeutic Medicine*.

[33] Niewczas M. A., Gohda T., Skupien J. (2012). Circulating TNF receptors 1 and 2 predict ESRD in type 2 diabetes. *Journal of the American Society of Nephrology*.

[34] Myśliwska J., Zorena K., Semetkowska-Jurkiewicz E., Rachón D., Suchanek H., Myśliwski A. (2005). High levels of circulating interleukin-10 in diabetic nephropathy patients. *European Cytokine Network*.

[35] Huang X. R., Kitching A. R., Tipping P. G., Holdsworth S. R. (2000). Interleukin-10 inhibits macrophage-induced glomerular injury. *Journal of the American Society of Nephrology*.

[36] Rodrigues K. F., Pietrani N. T., Sandrim V. C. (2015). Association of a large panel of cytokine gene polymorphisms with complications and comorbidities in type 2 diabetes patients. *Journal of Diabetes Research*.

[37] Stehouwer C. D. A., Stroes E. S. G., Hackeng W. H. L., Mulder P. G. H., Den Ottolander G. J. H. (1991). Von Willebrand factor and development of diabetic nephropathy in IDDM. *Diabetes*.

[38] Holzinger C., Weissinger E., Zuckermann A. (1993). Effects of interleukin-1, -2, -4, -6, interferon-gamma and granulocyte/macrophage colony stimulating factor on human vascular endothelial cells. *Immunology Letters*.

[39] Oggianu L., Lancellotti S., Pitocco D. (2013). The oxidative modification of von Willebrand factor is associated with thrombotic angiopathies in diabetes mellitus. *PLoS ONE*.

[40] Bernardo A., Ball C., Nolasco L., Moake J. F., Dong J.-F. (2004). Effects of inflammatory cytokines on the release and cleavage of the endothelial cell-derived ultralarge von Willebrand-factor multimers under flow. *Blood*.

